# The Promise of Agriculture Genomics

**DOI:** 10.1155/2017/9743749

**Published:** 2017-03-05

**Authors:** Wenqin Wang, Xuan H. Cao, Mihai Miclăuș, Jianhong Xu, Wenwei Xiong

**Affiliations:** ^1^Shanghai Jiaotong University, Shanghai, China; ^2^Leibniz Institute of Plant Genetics and Crop Plant Research, Gatersleben, Germany; ^3^National R&D Institute for Biological Sciences, Cluj-Napoca, Romania; ^4^Institute of Crop Science, Zhejiang Key Laboratory of Crop Germplasm, Zhejiang University, Zhejiang 310058, China; ^5^Montclair State University, Montclair, NJ, USA

With the fact of growing global population, changing climate, and environmental pressure, there is an urgent need to accelerate breeding novel crops with higher production, drought or heat tolerance, and less pesticide usage. Advances in genomics offer the potential to speed up the process of developing crops with promising agronomic traits. Agriculture genomics is the application of genomics in agriculture to improve the productivity and sustainability in crop and livestock production. With the combination of traditional and high-throughput sequencing platforms, there has been a tremendous increase in genomic resources available, including expressed sequence tags (ESTs), BAC end sequence, genetic sequence polymorphisms, gene expression profiling, whole-genome (re)sequencing, and genome-wide association studies. Given the emergence of genomic sequencing and expansion of bioinformatic tools, we are shifting from single gene study to whole-genome analysis, which offers a broader view of how all genes work together. Therefore, this special issue was organized, comprising eleven original research papers and three reviews aimed to highlight recent advances in (1) comparative genomics for plant breeding, (2) transcriptome analysis for plant breeding, (3) genotyping and marker-assisted breeding, and (4) recombinant DNA technology (Figure 1).

*Comparative Genomics for Plant Breeding*. To date, the genome sequences for more than 55 plant species (mainly model plants, such as* Arabidopsis*, rice, and maize) have been produced. The 1000-plant (one KP or 1 KP) initiative is underway. As a result, scientists are not totally dependent on genomic sequences of model plants. Almost every species-specific genome can be sequenced with affordable price and thus offer great opportunities for targeted crop breeding. Furthermore, genomics is playing a very crucial role in biodiversity conservation. Advanced genomics helps in identifying the segments of the genome responsible for adaptation. It can also improve our understanding of microevolution through a better understanding of natural selection, mutation, and recombination, as summarized in S. Khan et al.'s article. Understanding the structure, organization, and dynamics of genomes in plant species can provide insights into how genes have been adapted by natural and artificial selection to respond to environmental constraints and the potential of their manipulation for crop improvement.

Conventional breeding in agriculture is based exclusively on phenotypic selection. Until the availability of genomic sequence for model plants, comparative genomics approaches were successful for identifying homologues/orthologues or cloning species-specific genes by using sequence conservation or synteny from model plant systems. The group of D. T. Le surveyed the genomes of* Arabidopsis* and soybean for genes encoding Met-rich proteins (MRPs) based on sequence similarity. Genes encoding MRPs were classified into functional categories including RNA transcription, protein modification, and calcium signaling. It was found that MRPs were mainly responsible for drought and salinity stress in* Arabidopsis* and soybean.

Predicting gene function solely based on homology to others can sometimes be difficult. Thus, proteomics (the large-scale analysis of proteins) will greatly contribute to our understanding of gene function in the postgenomic era. The group of J. Lee used shotgun proteomic analysis to quantify the protein in* Brassica rapa* under drought treatment. Their results showed that the levels of proteins associated with photosynthesis were decreased while the proteins involved in catabolic processes and stress responses were increased, rendering their genes as potential targets for engineering drought resistance in plants.

*Transcriptome Analysis for Plant Breeding*. With numerous genome sequences being deposited into the public databases at an accelerating pace, it is still challengeable to translate sequence into function directly. In this context, additional efforts are needed to understand the gene structure and quantify its expression. Y. Kong et al. took such an approach by molecularly cloning, characterizing, and quantifying the gene expression of hemocyanin subunit 1 (*MnHc-1*) in oriental river prawn. The full-length cDNA of* MnHc-1* was 2,163 bp with a 2,028 bp open reading frame (ORF) encoding a polypeptide of 675 amino acids. The* MnHc-1* gene was expressed in the hepatopancreas, gill, hemocytes, intestine, ovary, and stomach, with the highest level in the hepatopancreas. Their investigation indicated that the* MnHc-1* expression can be influenced by dietary copper and the hemocyanin may potentially participate in antibacterial defense. The method provides the foundation for further studies to understand gene function using loss-of-function mutant phenotype.

In the absence of the complete genome sequence, transcriptome analysis would improve our understanding of gene function. A global transcriptome study unveils the gene responses to a particular biological condition at the genome level. For example, aluminum and acid combination is the main factor limiting plant growth and crop production worldwide. P. Zhou et al. used microarray data in alfalfa seedlings to investigate how acid soils and aluminum toxicity impact the global gene expression. The main functional categories involved in phytohormone regulation, reactive oxygen species, and transporters were enriched after aluminum stress in alfalfa. Their results contribute towards understanding the key regulatory genes and pathways that would be advantageous for improving crop production not only in alfalfa but also in other crops under aluminum-acid stress. The transcript levels of MRP-coding genes under normal and stress conditions in* Arabidopsis* and soybean were also studied by microarray indicating that MRPs participate in various vital processes of plants under normal and stress conditions.

RNA-Seq, also called whole transcriptome shotgun sequencing, uses next-generation sequencing (NGS) to reveal the presence and quantity of RNA in a biological sample at a given time, which is a more sensitive and accurate way to investigate genome-wide gene expression than microarray. Using RNA-Seq in rainbow trout with high and low carcass fat content, the group of B. Wang identified 1,694 differentially expressed transcripts involved in lipid metabolism, such as L-FABP, adiponectin, PPAR-*α*, PPAR-*β*, and IGFBP1a. Their findings also indicated that PPAR-*α* and PPAR-*β* could be used as molecular markers for fat storage in rainbow trout liver. 

*Genotyping and Marker-Assisted Breeding*. In addition to the generation of reference genome, high-throughput sequencing technology has facilitated resequencing of genomes of the same species but different accessions to identify genomic variation. The genotyping platforms have been used to generate large-scale marker segregation data on mapping populations and have led to comprehensive genetic maps. The genome sequence allows us to identify genome-wide molecular markers including functional markers, candidate genes, and predictive markers for breeding.

Duckweeds are promising plants to clean wastewater and to be digested into renewable biofuel. It was reported that different ecotypes of the same duckweed species exhibit a variety of biochemical and physiological properties. Developing sensitive markers to select desirable ecotypes is critical in plant breeding. The group of H. Zhao designed three molecular markers, PCR-amplified their products, and ran them through high-resolution capillary electrophoresis. Eleven haplotypes were found both in* Spirodela polyrhiza* and in* Landoltia punctata*. The marker system with multiple sequence polymorphisms is sensitive to intraspecies discrimination compared with interspecies identification and thus will promote large-scale identification at the ecotype level.

Cowpea is one of the most important legume crops in the world. It is also a major food crop in Africa, Latin America, and India because of its high protein content. Genetic diversity is the greatest resource for plant breeders to select lines that could potentially enhance food quality and quantity. The group of E. N. Wamalwa evaluated genetic diversity in 19 cowpea accessions from the Kenyan national gene bank, which they classified into two major groups. Breeders can now cross genetically distant accessions from those two groups for the improvement of cowpea crop, harnessing the power of heterosis.

Molecular markers such as simple sequence repeats (SSR) and single nucleotide polymorphism (SNP) from genomic and transcriptomic studies are great resources in plant breeding, used for trait dissection and for enhancing precision in selecting functional genes. Tomato is a thermophilic vegetable and is sensitive to low temperature. To map QTLs conferring cold tolerance in tomato, the group of H. Chen developed 120 SSR markers from a population of 146 RILs (Recombinant Inbred Lines) that was derived from a cross between a cold-sensitive cultivated* Solanum lycopersicum* and a cold-tolerant wild* Solanum pimpinellifolium*. The study resulted in nine QTLs providing references for further fine mapping of cold tolerance. Meanwhile, the polymorphic markers that have been developed can be used in selecting desirable traits and aid in developing new tomato varieties by marker-assisted breeding. The group of T. D. Khanh analyzed five typical rice landraces including three* indica* and two* japonica* by using 30-fold coverage of short paired-end reads from NGS. Compared with reference genomes, they determined more than two million SNPs and INDELs that would provide informational resources to help further marker-assisted selection in rice breeding programs.

Genome-wide association study is another method to find the relationship between molecular markers and QTL based on linkage disequilibrium. Association mapping on founder parents and their derivatives can find some important QTL and favorable allelic variations, which can be further used for marker-assisted selection to produce more favorable varieties. The group of H.-X. Ma screened Ningmai 9 wheat and 117 of its derivatives for their solvent retention capacity (SRC) and associated this phenotypic trait with 29 QTL markers. Their study was aimed at improving breeding soft wheat flour for cookie quality. 

*Recombinant DNA Technology*. Recombinant DNA technology is a milestone in plant science and crop breeding that can help to design almost any desirable characteristic by controlled targeted gene expression. The team of H. Hou reviewed the history, the current research progress, and applications of recombinant DNA technology. They summarize that the genetically modified plants have been shown to possess improved resistance to harmful agents, enhanced product yield, and increased adaptability for abiotic stress.

One of the most powerful tools of recombinant DNA technology is CRISPR-Cas9-induced genome editing. H. X. Cao et al. overviewed the CRISPR-Cas9 system and the major technical advances for manipulation of model and crop plant genomes. They also discussed the future perspectives of CRISPR-Cas technology in molecular plant breeding. It is believed that the promise of next green revolution with new crops meeting long-standing requests is soon to be achieved greatly aided by the rapid development of CRISPR-Cas technology.

Genetically modified crops are plants that had their DNA genetically modified using recombinant DNA technology. For example, transgenic maize (WL-73) plants overexpressing the* BcWRKY1* gene were generated by* Agrobacterium*-mediated transformation, which were able to resist 300 mM NaCl stress. Still, the crop safety needs to be further evaluated. X. Zeng et al. measured the effects on rhizosphere soil in terms of enzyme activities, physicochemical properties, and microbial populations between the genetically modified maize, overexpressing the* BcWRKY1* gene, and nontransgenic maize. They reported that salinity-tolerant transgenic maize had no adverse impact on soil rhizosphere during a period of three consecutive years, which paves the way for further commercialization.

## Figures and Tables

**Figure 1:**
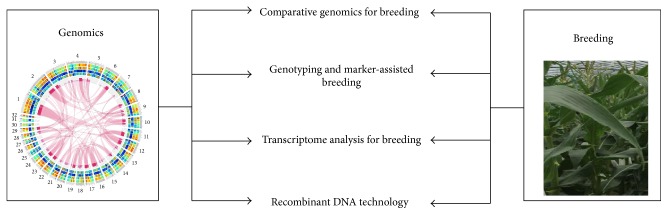
Advances in this special issue.

